# Experimental and Finite Element Analysis of Compressive Strength and Diametral Tensile Strength of Luting Cement: An In Vitro Study

**DOI:** 10.7759/cureus.64658

**Published:** 2024-07-16

**Authors:** Sazan S Saleem, Sazgar M Sabir, Kareem A Abdulla

**Affiliations:** 1 Department of Pedodontics, Orthodontics, and Preventive Dentistry, College of Dentistry of Hawler Medical University, Erbil, IRQ; 2 Department of Mechanical and Mechatronics, College of Engineering, Salahaddin University Erbil, Erbil, IRQ

**Keywords:** glass ionomer cement, diametral tensile strength, compressive strength, luting cement, finite element analysis

## Abstract

Background

Strength parameters greatly influence the selection of luting agents. This study compared the compressive and diametral tensile strengths (DTS) of three luting cements.

Materials and methods

Three luting cements, conventional glass ionomer (CGI), resin-modified glass ionomer (RMGI), and resin cement (RC), were tested for compressive strength and DTS. Forty-two standardized specimens were prepared, measuring 4 mm by 6 mm for compressive tests and 6 mm by 3 mm for diametral tensile tests. The luting materials were prepared according to the manufacturers' instructions.

Result

Experimental mean compressive and diametral strengths and standard errors were calculated for each luting agent (n = 10). Analysis of variance was computed (p < 0.05), and multiple comparison tests were performed. RC showed significantly higher compressive strengths and DTS among the three tested luting cements, while the CGI showed the least. The results obtained by finite element analysis (FEA) for both tests closely matched the experimental results.

Conclusion

In this study, it was concluded that the mean compressive strength and DTS values of all three luting cements were significantly different. The resin luting cement exhibited the highest compressive strength and DTS, while the CGI exhibited the least.

## Introduction

In recent years, there has been a significant increase in the number of individuals experiencing partial edentulism, resulting in a growing demand for fixed partial dentures [1]. Dental luting cements play a vital role in establishing a durable bond between dissimilar materials, acting as the intermediary between a fixed prosthesis and the supporting prepared tooth structure. A variety of materials are commercially available as luting agents for dental purposes. These materials must withstand mechanical stresses and challenging oral environmental conditions while maintaining integrity and effectively transferring stresses from crowns and fixed partial dentures to the tooth structure. Additionally, dental luting cements serve as a barrier against microbial leakage, sealing the interface between the tooth and the restoration [2, 3].

A diverse range of provisional and long-term cements is available, each characterized by its unique chemical composition, properties, and clinical applications. Making informed decisions and applying cement accurately in clinical practice requires a thorough evaluation and understanding of the material composition, bonding mechanisms, and interactions with other restorative materials [4, 5].

Water-based luting cements, such as glass-ionomer and resin-modified glass-ionomer (RMGI), are prominent among the current materials used. These possess fluoride release properties and have gained popularity due to their exceptional wettability and bonding capabilities with enamel and dentin. In contrast, resin cements (RCs), which share chemical affinities with composite resins, offer optimal strength to both the tooth and indirect restoration when combined with dental adhesives [5].

Understanding the properties of luting materials and their clinical indications is essential to ensure the quality of cementation [6]. The selection of luting agents for cementing retainers in fixed partial dentures is significantly influenced by strength parameters. High compressive strength is crucial to withstand masticatory forces. Additionally, many luting agents are inherently brittle and prone to tensile failure [2, 5]. Stronger cements contribute to more uniform stress distribution, reducing the likelihood of tensile or compressive failure and enhancing the potential for clinical success. Compressive and tensile strength are critical indicators for functional evaluation and prerequisites of clinical application. The compressive strength of dental resin luting cements significantly influences the success and durability of dental restorations [2]. Therefore, there is a pressing need to investigate and compare the strengths of currently used luting agents.

Understanding the properties of luting materials and their clinical indications is essential to ensure the quality of cementation [6]. The selection of luting agents for cementing retainers in fixed partial dentures is significantly influenced by strength parameters. High compressive strength is crucial to withstand masticatory forces. Additionally, many luting agents are inherently brittle and prone to tensile failure [2, 5]. Stronger cements contribute to more uniform stress distribution, reducing the likelihood of tensile or compressive failure and enhancing the potential for clinical success. Compressive and tensile strength are critical indicators for functional evaluation and prerequisites of clinical application. The compressive strength of dental resin luting cements significantly influences the success and durability of dental restorations [2]. Therefore, there is a pressing need to investigate and compare the strengths of currently used luting agents.

Finite element analysis (FEA) is a computational method used to assess stresses and deformations in structures. Originally developed to address complex structural issues in civil and aeronautical engineering, FEA breaks down intricate geometries into a set of "finite" dimensions, creating a meshed model of the object under analysis. Each finite element is defined by nodes, and equilibrium equations for every degree of freedom at each node are established based on its internal strain function. The analysis begins by partitioning the complex body into finite, simpler elements through a mesh generation process controlled by the user. For static structural analysis of linear isotropic materials, parameters such as density, elasticity modulus, and Poisson's ratio are assigned to each element. Subsequently, loads are applied at specific nodes or groups of nodes, and constraints are imposed by fixing certain nodal displacements to predetermined values [7].

Understanding how applied loads transfer to stress and the distribution patterns of various types of stress within tested specimens are fundamental concepts in dental biomechanics [8]. Achieving this objective would be challenging without the assistance of a robust computer program such as FEA. To address this, the study aims to assess the compressive strengths and diametral tensile strength (DTS) of the following luting agents: glass ionomer cement (GC Fuji I), RMGI cement (GC Fuji Plus), and RC (G-CEM ONE), utilizing both experimental and numerical methods (FEA). This comparative analysis will offer valuable insights into the performance and suitability of these luting agents for clinical applications.

## Materials and methods

This study involved the preparation of a total of 42 specimens (n = 42) using cylindrical metal molds, adhering to standard dimensions prescribed for each specific test. The specimens were categorized into three groups based on the materials used in the study: CGI, RMGI, and RC.

**Figure 1:**
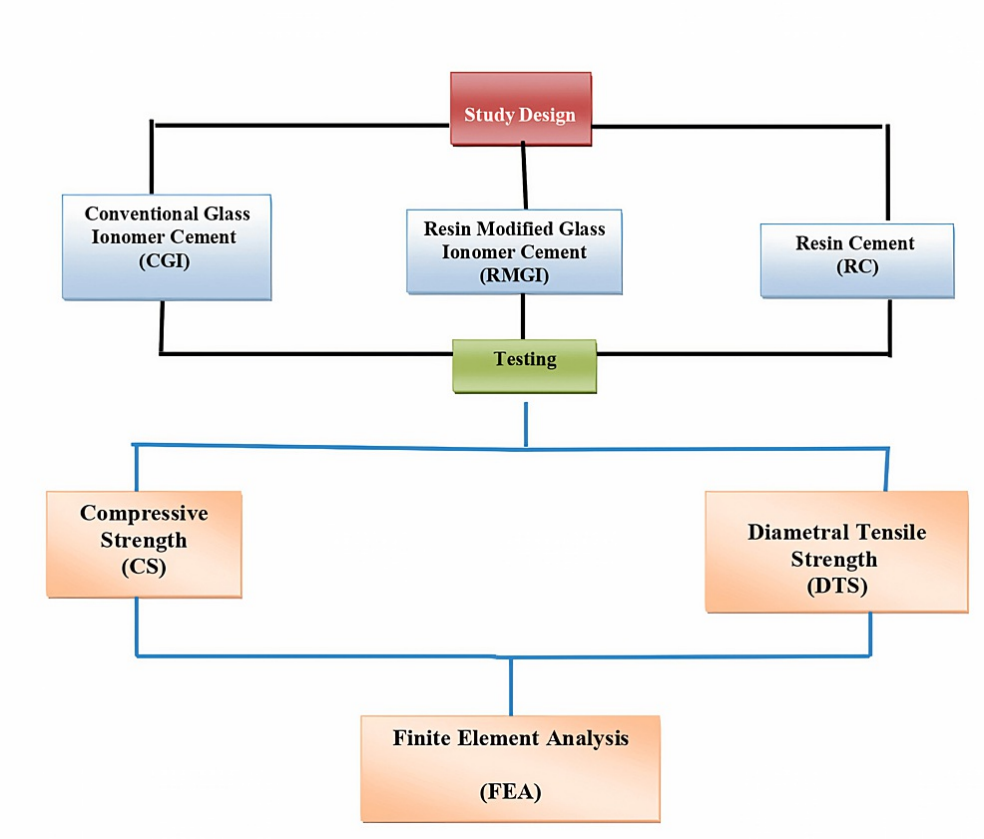
A schematic diagram showing sample grouping. Image credits: Saleem SS et al.

Subsequently, each of these groups was further subdivided into two subgroups based on the tests they underwent, namely, compressive test and DTS. FEA was then conducted for the specimens to determine the numerical results of the compressive and diametral tensile strength. The details of the materials used and the study design are outlined in Table 1 and Figure 1, respectively.

**Table 1:** Commercial names, classifications, manufacturers, and properties of the luting materials used in the study.

Materials used in this study	Code	Materials classification	Materials manufacturers	Curing mode	Delivery system
GC FUJI I	CGI	Conventional glass ionomer	GC Corporation, Japan	Chemically cured	Capsule mixing/delivery
GC FUJI PLUS	RMGI	Resin-modified glass ionomer	GC Corporation, Japan	Chemically cured	Capsule mixing/delivery
G-CEM ONE resin cement	RC	Self-adhesive resin cement	GC Corporation, Japan	Light cured	Twin tube with automix tip

Preparation of the specimens

The preparation of the specimens involved the use of two metal molds, each tailored to specific dimensions for the intended tests. The entire process adhered meticulously to the manufacturer's instructions for each material. The procedure commenced with the metal mold being placed onto a glass plate.

The specimens for the compressive strength test were crafted in strict adherence to ISO 9917 specifications [9]. For the DTS test, dimensions of 6.0 mm in diameter and 3.0 mm in height were employed, following guidelines outlined by the American Dental Association (ADA) [10].

To prepare the specimens of encapsulated cements (groups 1 and 2), the capsules were activated using an amalgamator, triturated according to the manufacturer's guidelines, and loaded into a capsule applicator. The blended cement was then extruded through the nozzle of the capsule directly into the mold. Subsequently, an additional glass plate was pressed onto the slightly overfilled mold's open end to remove excess materials.

For resin cement specimens, the cement was supplied in dual-barrel syringes equipped with single-use automix tips. The two components were thoroughly mixed within the syringe barrels using a spiral mixer, then dispensed directly into the mold hole. The application was performed in layers, each with a thickness of 2 mm. Subsequently, the cement was irradiated for the recommended exposure time through a mylar strip using an LED curing unit with a wavelength range of 440-480 nm and an emitting light intensity of 1500 mW/cm², employing an Elipar S10 (stainless steel Model with the light tip of 10 mm in diameter).

**Figure 2:**
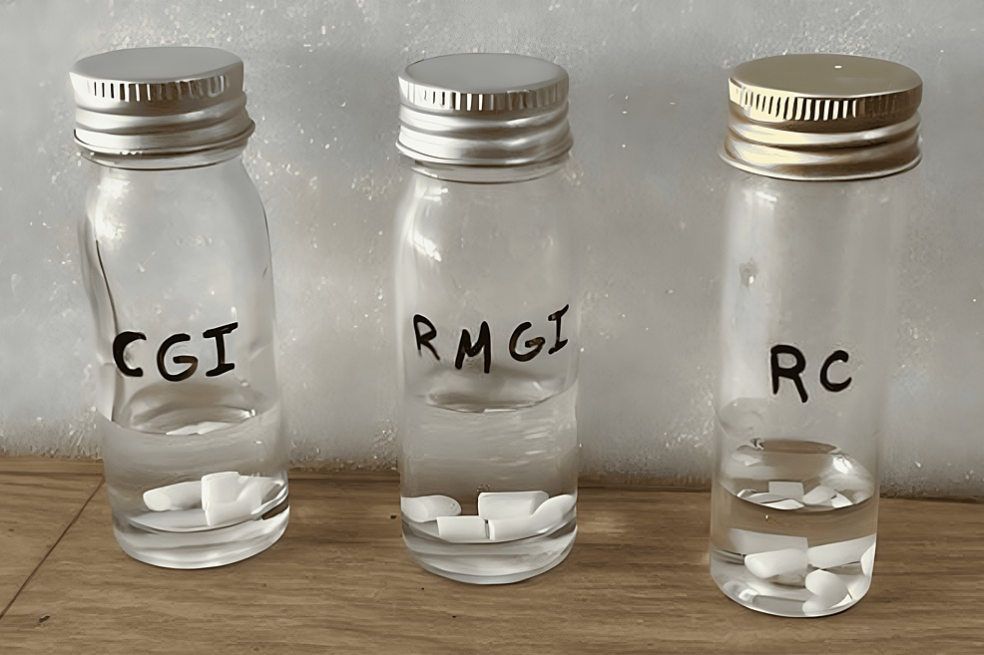
Specimens stored in test tube. CGI: Conventional glass ionomer; RMGI: Resin-modified glass ionomer; RC: Resin cement.

Finally, the specimens were extracted from the mold and immersed in distilled water at 37°C for 24 hours (Figure 2). Accurate measurements of the specimen dimensions were recorded using a micrometer with a precision of 0.001 mm.

Experimental method

Compressive Strength Test

**Figure 3:**
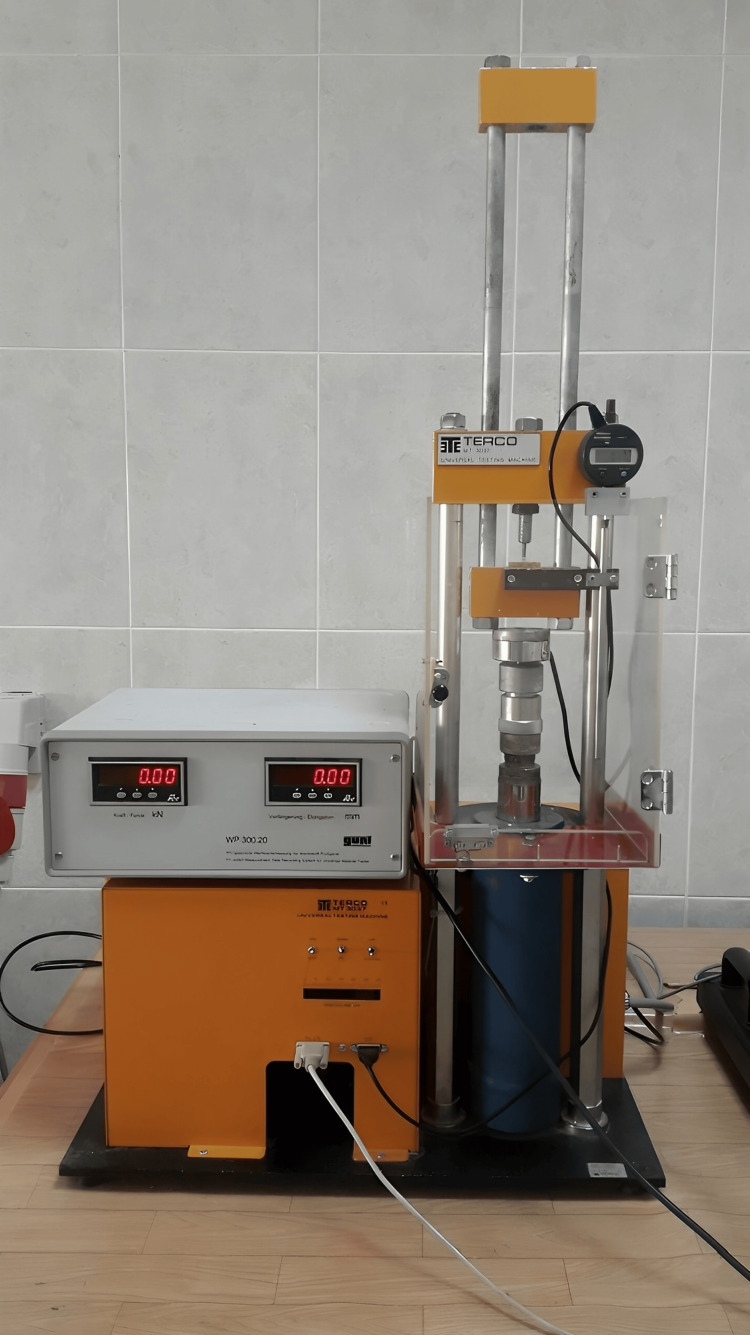
Universal testing machine (ETE TERCO MT 3037). Image credits: Saleem SS et al.

**Figure 4:**
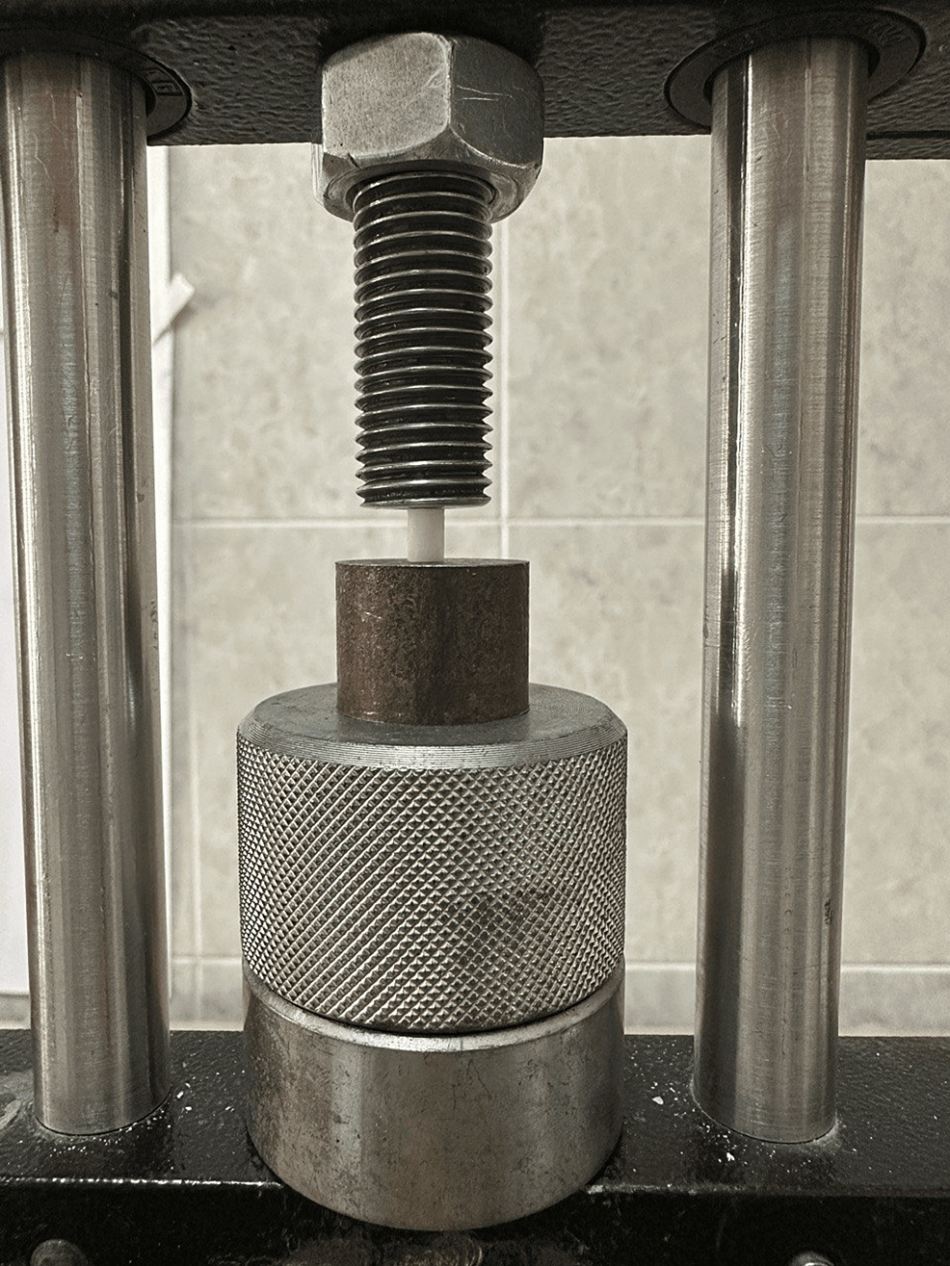
Specimen under loading for compressive strength. Image credits: Saleem SS et al.

After 24 hours of immersion in distilled water at 37°C, the samples underwent a compressive strength test using a universal testing machine. The cylindrical specimens were vertically positioned along their long axis between the flat surfaces of metal plates attached to a jig connected to the universal testing machine (Figures 3-4).

The loading proceeded at a crosshead speed of 1 mm/min until fracture occurred. The maximum applied load (P) causing specimen fracture was observed on the monitor, recorded (Figure 3), and calculated using the formula:

CS = 4P / π D,

where P represents the maximum applied load in Newtons (N), and D is the measured diameter of the sample in millimeters (mm).

Diametral Tensile Strength

Brittle materials such as cement or ceramics lack a yield point, and thus, their rupture strength is equivalent to their ultimate strength. These materials exhibit fracture under low tension, making the evaluation of tensile strength unreliable due to their low cohesive condition. An alternative approach involves assessing tensile strength through compressive testing, which offers a relatively straightforward and reproducible method. This is achieved through a diametral compression test for tension, also known as indirect tension. In this test, a disc sample is compressed diametrically to introduce tensile stress in the material along the plane of force application during the test [11].

**Figure 5:**
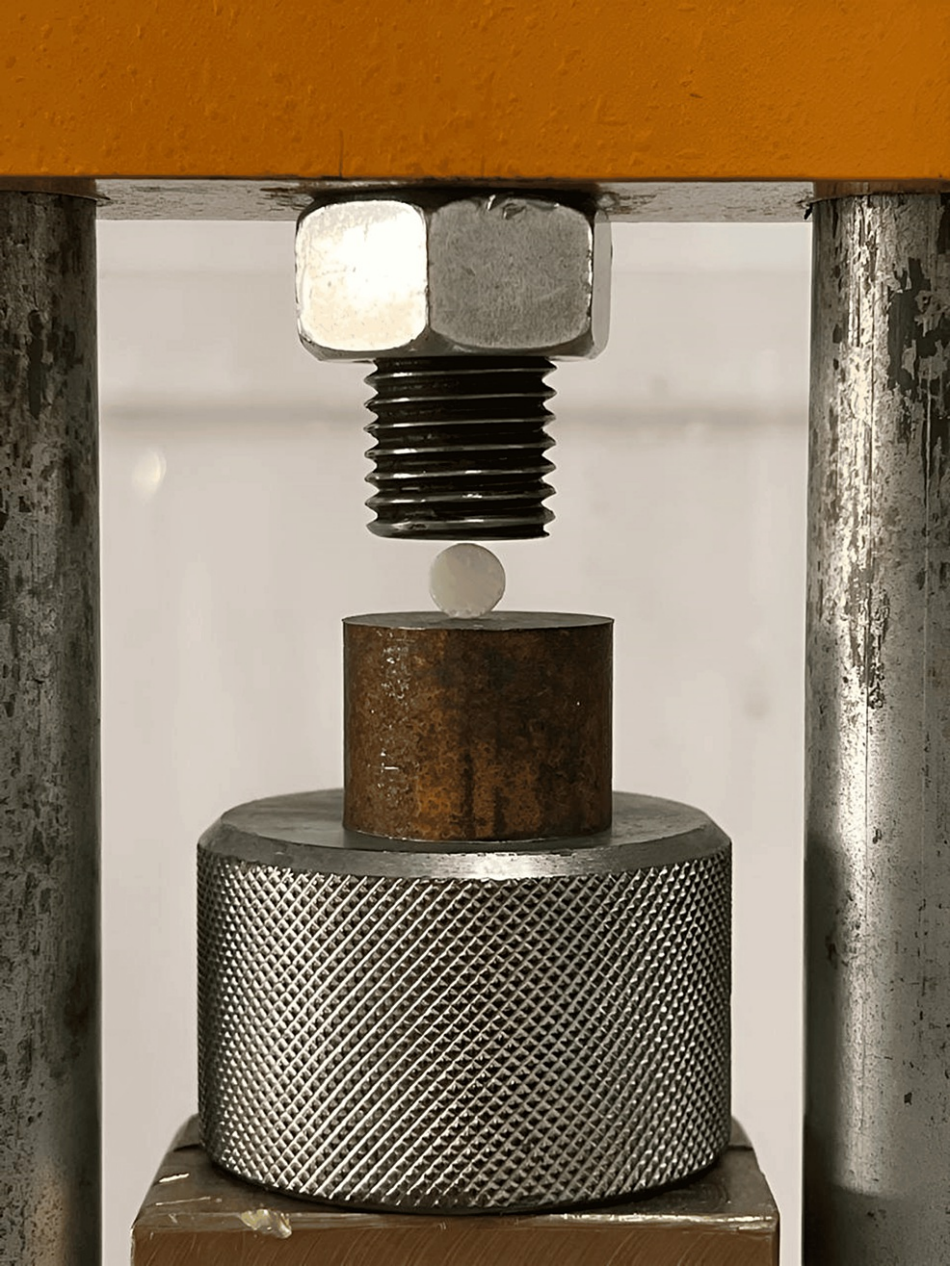
Specimen under loading for diametral tensile strength (DTS). Image credits: Saleem SS et al.

After preparing the specimens and 24 hours of storage in water, they were positioned with their flat ends perpendicular to the platens, as illustrated in Figure 5.

The specimens were placed in the Instron universal testing machine to ensure that the load was applied across the diameter of the specimens. During the test, the specimens were recorded at a crosshead speed of 0.1 mm/minute, and the DTS was calculated using the formula:

T = 2P/πDL, where P represents the maximum applied load (N), D is the measured diameter of the sample (mm), and L is the measured length of the sample (mm) [12,13].

Numerical method

FEA Loading and Boundary Conditions

The elastic properties of a material, including the Elastic (Young’s) modulus, Shear modulus (modulus of rigidity), Poisson’s ratio, and Bulk modulus, play a crucial role in characterizing its behavior under load. These properties enable the correlation of deformation occurring in solid structures subjected to external forces.

According to Hooke’s law, the relation between stress (σ) and strain (ε) in a linear elastic isotropic material is expressed by the following equations:

$\epsilon x=\sigma x-v\sigma y\div E$

$\epsilon y=\sigma y-v\sigma x\div E$

$\epsilon z=-v ( \sigma y+\sigma x)\div E$

For isotropic materials, the elastic properties are related as follows:

$G=E\div 2(1+v)$

$K=E\div 3(1-2v)$

where:

E: Elastic (or Young’s) modulus;

G: shear modulus (or modulus of rigidity);

ν: Poisson’s ratio;

K: Bulk modulus.

σx: stress component on the horizontal axis;

σy: stress component on the vertical axis.

Hence, two independent parameters are sufficient to identify the stiffness of a solid: Young’s Modulus and Poisson's ratio.

A finite element model was constructed using ANSYS Workbench (version 2020 R1) to simulate the behavior of soft tissue materials. Models were developed for various suggested designs of these materials to investigate their performance under different conditions. This enabled the examination of each design's behavior, facilitating the optimization of design parameters. The objective was to identify the most suitable material with reduced stress levels, improved durability, and the ability to achieve the desired resection rate. FEA was conducted on the specimens to obtain numerical results for compressive and diametral tensile strength [14].

The mechanical properties obtained from experimental testing were utilized as input data for numerical analysis. The ANSYS Workbench (version 2020 R1) was employed to conduct numerical modeling of cylindrical shapes: a height (h) of 6 mm and a diameter (d) of 4 mm for compressive strength, and a height of 3 mm and a diameter of 6 mm for DTS.

For enhanced accuracy, the specimens were subdivided into 13,050 3D small elements, each consisting of simple shapes interconnected by 14,508 nodes following meshing. Consequently, stress calculations were performed for all small elements, enabling a comprehensive assessment of the stress-strain state across the entire specimen.

## Results

Compressive strength

The results of the descriptive statistical test for the compressive strength of the tested materials are presented in Table 2. The highest mean value of compressive strength was observed in the RC group (189.228 ± 16.8283), which was significantly different from the other groups, as indicated by different letters in Table 2. Following RC, the RMGI group had a mean compressive strength of 81.081 ± 10.9981. In contrast, the CGI group exhibited the lowest mean compressive strength value (55.078 ± 4.8187), which was also statistically significantly different from the other groups.

**Table 2:** Descriptive statistics (mean values, SDs, standard errors, and 95% CIs) for compressive strength. Note: The letters indicate that different letters had significant difference.. CGI: Conventional glass ionomer; RMGI: Resin-modified glass ionomer; RC: Resin cement.

Groups	N	Mean	SD	Std. Error	95% Confidence Interval for Mean		Minimum	Maximum
					Lower Bound	Upper Bound		
CGI	7	55.078 a	4.8187	1.8213	50.6214	59.5345	50.160	64.390
RMGI	7	81.081 b	10.9981	4.1569	70.9101	91.2533	70.070	102.710
RC	7	189.228 c	16.8283	6.3605	173.6647	204.7918	160.040	207.000
Total	21	108.46267	60.5901	13.221	80.8823	136.0429	50.160	207.000

The statistical analysis of the data using an ANOVA test indicated a highly significant difference (P < 0.000) in compressive strength values among the tested groups, as depicted in Table 3.

**Table 3:** ANOVA test for difference among groups for the compressive test.

Source of Variation	Sum of Squares	df	Mean Square	F	P-value
Between Groups	70859.071	2	35429.535	248.703	0.000
Within Groups	2564.229	18	142.457		
Total	73423.300	20			

Multiple Comparison Tests (LSD) were conducted, revealing significant differences among the tested materials, as illustrated in Table 4. The discrepancy between the RC group and the other two groups is highly significant (P = 0.000). Furthermore, the disparity between the RMGI and CGI groups is significant (P < 0.05). Table 4 supports Table 2, where distinct letters denote each group.

**Table 4:** Multiple comparison (LSD) test for the compressive strength of tested materials. CGI: Conventional glass ionomer; RMGI: Resin-modified glass ionomer; RC: Resin cement; LSD: Least significant difference.

Test	(I) factor	(J) factor	Mean Difference	Std. Error	95% Confidence Interval		P-value
					Lower Bound	Upper Bound	
LSD	CGI	RMGI	-26.0037^*^	6.3798	-42.2860	-9.7213	0.002
		RC	-134.1502^*^	6.3798	-150.4326	-117.8679	0.000
	RMGI	CGI	26.0037^*^	6.3798	9.72138	42.2860	0.002
		RC	-108.1465^*^	6.3798	-124.4289	-91.8642	0.000
	RC	CGI	134.15028^*^	6.3798	117.8679	150.4326	0.000
		RMGI	108.14657^*^	6.3798	91.8642	124.4289	0.000

The experiments were conducted using the INSTRON ### 600 KN universal testing machine. The objective of compression testing is to assess the behavior of a material under compressive loads by measuring fundamental variables such as strain, stress, and deformation. Through compression testing, parameters including compressive strength, yield strength, ultimate strength, elastic limit, and elastic modulus, among others, can be determined. Table 5 presents the properties of the luting materials obtained from the compression test experiments.

**Table 5:** The properties of the luting materials used in the study. CGI: Conventional glass ionomer; RMGI: Resin-modified glass ionomer; RC: Resin cement.

Materials of luting	RC	RMGI	CGI
Young Modulus (MPa)	26.00	23.59	20.00
Poisson’s Ratio	0.25	0.25	0.25
Bulk Modulus (MPa)	17.33	15.33	13.33
Shear Modulus (MPa)	10.40	9.2	8
Density (Kg/m^3^)	1500	1500	1250

DTS

The results of the descriptive statistical test for the DTS of the tested materials are presented in Table 6. The highest mean DTS value was observed in the RC group (13.1943 ± 2.12429), which was significantly different from the other groups, as indicated by the distinct letters in Table 6. This was followed by the RMGI group, which had a DTS of 5.7429 ± 1.14064. The CGI group exhibited the lowest mean DTS value (2.6529 ± 0.57462), which was also statistically significantly different from the other groups.

**Table 6:** Descriptive statistics (mean value, SD, standard errors, and 95% CIs) for diametral tensile strength (DTS). Note: The letters indicate that different letters had significant difference CGI: Conventional glass ionomer; RMGI: Resin-modified glass ionomer; RC: Resin cement.

Groups	N	Mean	SD	Std. Error	95% Confidence Interval for Mean		Minimum	Maximum
					Lower Bound	Upper Bound		
CGI	7	2.6529 a	0.57462	0.21719	2.1214	3.1843	1.95	3.36
RMGI	7	5.7429 b	1.14064	0.43112	4.6879	6.7978	4.25	7.61
RC	7	13.1943 c	2.12429	0.80291	11.2296	15.1589	10.97	17.17
Total	21	7.1967	4.73277	1.03277	5.0423	9.3510	1.95	17.17

Statistical analysis of data using the ANOVA test revealed a highly statistically significant difference (P < 0.000) in DTS values among the tested groups, as shown in Table 7.

**Table 7:** ANOVA test for difference among groups for the DTS. DTS: Diametral tensile strength.

ANOVA					
DTS					
	Sum of Squares	df	Mean Square	F	P-value
Between Groups	411.118	2	205.559	100.373	.000
Within Groups	36.863	18	2.048	-	-
Total	447.981	20	-	-	-

Multiple comparison tests (LSD) revealed significant differences among the tested materials, as detailed in Table 8. The difference between the RC group and the other two groups is highly significant (P=0.000). Furthermore, the difference between the RMGI group and the CGI group is significant (P<0.05). Table 8 corroborates the findings in Table 6, where different letters are used to distinguish each group.

**Table 8:** Multiple comparison tests (LSD test) for the DTS of tested materials. DTS: Diametral tensile strength; CGI: Conventional glass ionomer; RMGI: Resin-modified glass ionomer; RC: Resin cement; LSD: Least significant difference.

Multiple Comparisons						
Dependent Variable: DTS						
LSD						
(I) factor	(J) factor	Mean Difference (I-J)	Std. Error	Sig.	95% Confidence Interval	
					Lower Bound	Upper Bound
CGI	RMGI	-3.09000^*^	.76494	.001	-4.6971	-1.4829
	RC	-10.54143^*^	.76494	.000	-12.1485	-8.9344
RMGI	CGI	3.09000^*^	.76494	.001	1.4829	4.6971
	RC	-7.45143^*^	.76494	.000	-9.0585	-5.8444
RC	CGI	10.54143^*^	.76494	.000	8.9344	12.1485
	RMGI	7.45143^*^	.76494	.000	5.8444	9.0585
*. The mean difference is significant at the 0.05 level.						

Numerical analysis results (FEA)

Compressive Strength (CS)

**Figure 6:**
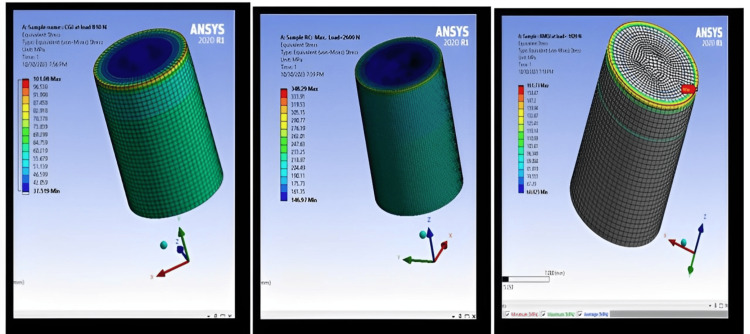
Compression test by FEA using ANSYS of three kinds of luting cement. FEA: Finite Element Analysis; ANSYS: Analysis System.

The properties obtained by FEA for each type of material are shown in Figure 6.

All three types of luting cement were modeled and simulated under surface loading applied to the top face of the specimens, replicating the conditions applied during testing using FEA. The resulting stress field and stress distribution within the material body are depicted in Figure 6, showing the transition from red to blue in the stress fields. This transition indicates an increase in the value of the vertical compressive stress, reaching its maximum at the edge of the bottom surface of the specimen. The compressive stress for CGI is 101.08 MPa at a load of 810 N, for RMGI it is 161.73 MPa at a load of 1020 N, and for RC it is 348.29 MPa at a load of 2600 N. The results obtained by FEA closely match the experimental results.

Diametral Tensile Strength (DTS)

**Figure 7:**
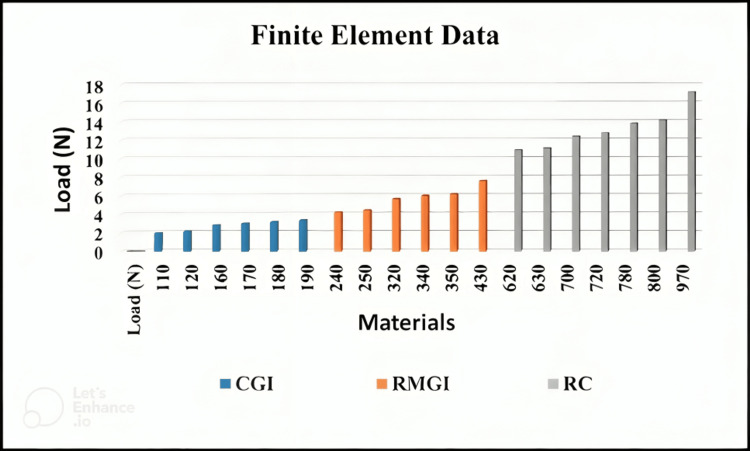
Bar chart for the DTS by FEA using ANSYS for three types of luting cement. FEA: Finite element analysis; ANSYS: Analysis system; DTS: Diametral tensile strength; CGI: Conventional glass ionomer; RMGI: Resin-modified glass ionomer; RC: Resin cement.

Figure 7 illustrates that sample RC exhibits greater stress strength (DTS) compared to RMGI and CGI samples, aligning with the experimental results. Sample RC requires a significantly higher stress force than RMGI and CGI samples to reach the breaking point.

**Figure 8:**
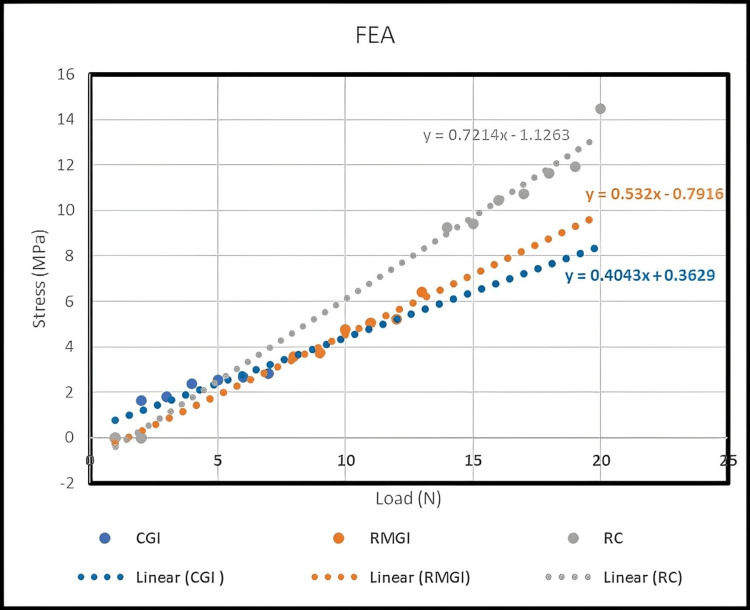
Relation between stress from DTS and load, analysed by FEA using ANSYS. FEA: Finite element analysis; ANSYS: Analysis system; DTS: Diametral tensile strength; CGI: Conventional glass ionomer; RMGI: Resin-modified glass ionomer; RC: Resin cement.

The results of FEA for DTS for each material are shown in Figure 8. This figure illustrates the relationship between applied load and stress (DTS). The slope value of 0.72, which exceeds those of 0.53 and 0.40, indicates that sample RC exhibits higher resistance force and stress compared to RMGI and CGI. Conversely, the slope of 0.40 indicates poor resilience for sample CGI.

**Figure 9:**
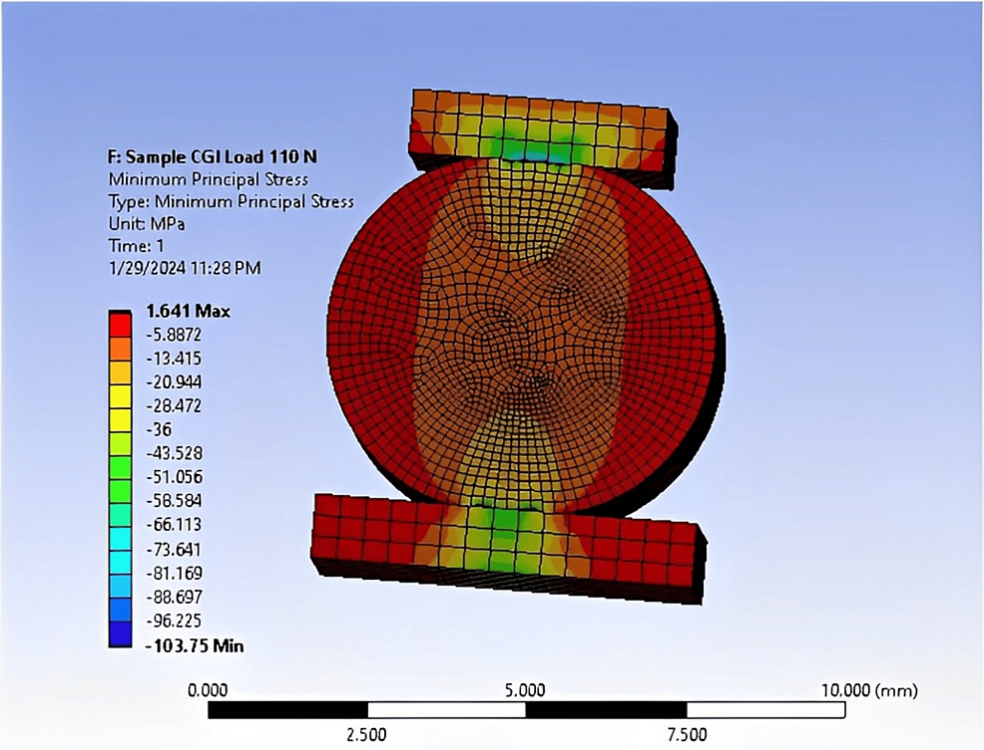
FEA of a CGI sample showing 1.64 MPa at a load of 110 N, conducted using ANSYS software. FEA: Finite element analysis; ANSYS: Analysis system; DTS: Diametral tensile strength; CGI: Conventional glass ionomer.

**Figure 10:**
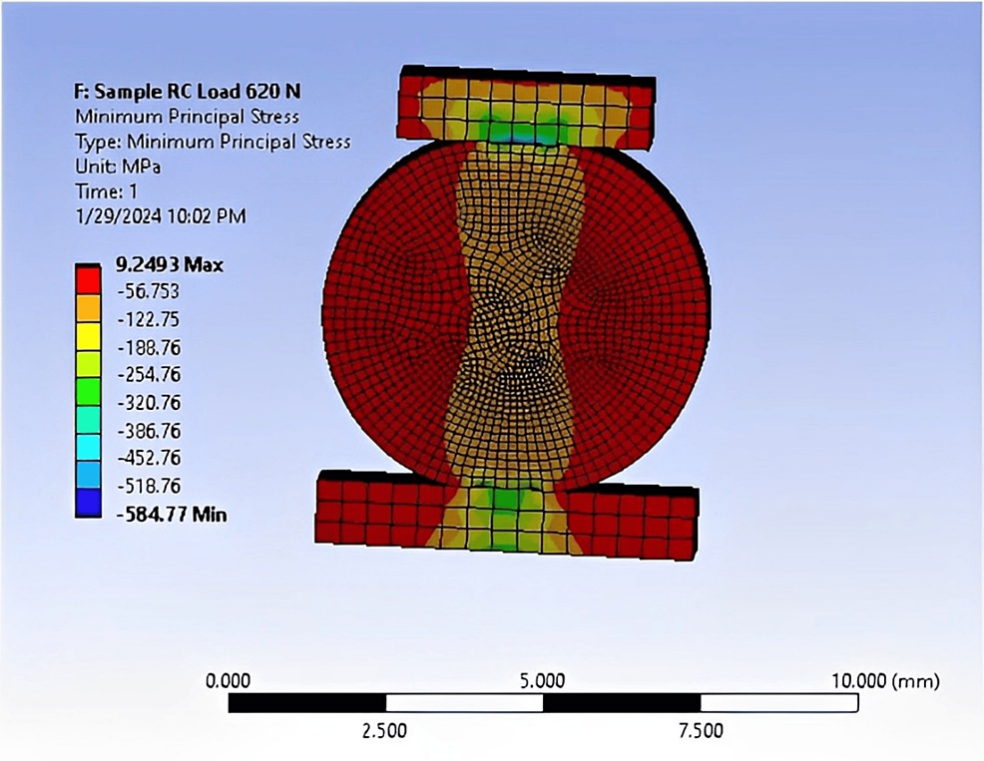
FEA of an RC sample showing 9.24 MPa at a load of 620 N, conducted using ANSYS software. FEA: Finite element analysis; ANSYS: Analysis system; RC: Resin cement.

**Figure 11:**
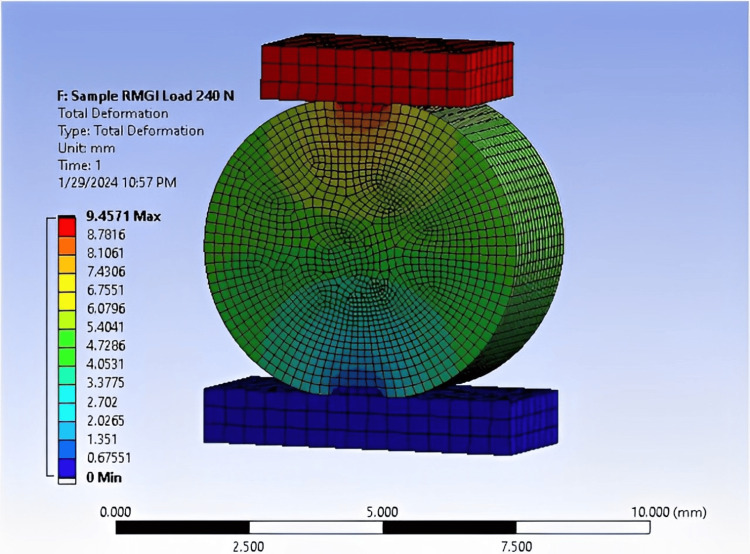
FEA of an RMGI sample showing 9.45 MPa at a load of 240 N, conducted using ANSYS software. FEA: Finite element analysis; ANSYS: Analysis system; RMGI: Resin-modified glass ionomer.

When conducting the practical test of the results, we sought to confirm them with numerical analysis. This confirmation is depicted in Figures 9-11.

The DTS value for RC (9.24 MPa at a 620 N load) closely aligns with experimental testing, with a relatively small error rate of 15%. Similarly, the DTS value for CGI (1.64 MPa at a 110 N load) also exhibits a 15% error rate. For RMGI, the DTS value is 9.45 MPa at a load of 240 N, with a slightly higher error rate of 15.7% (Table 9). These stress values highlight the differences in strength among the three samples. For instance, the RC sample withstood a force of 620 N at a stress of 9.24 MPa, indicating its robustness. In comparison, the RMGI sample withstood a load below 240 N but achieved a stress of 9.45 MPa, demonstrating its strength relative to the applied force. Conversely, the CGI model withstood a force of 110 N at a stress of 1.64 MPa, indicating its lower strength compared to the other materials. Based on this comparison, the RC model emerges as the optimal choice due to its superior performance under load.

**Table 9:** Experimental and FEA results of DTS. FEA: Finite element analysis; ANSYS: Analysis system; DTS: Diametral tensile strength; CGI: Conventional glass ionomer; RMGI: Resin-modified glass ionomer; RC: Resin cement.

	Experimental			FEA (ANSYS)					
Sample	CGI	RMGI	RC	CGI	RMGI	RC	CGI	RMGI	RC
Load (N)	DTS(Mpa)	DTS (Mpa)	DTS(Mpa)	DTS (Mpa)	DTS (Mpa)	DTS (Mpa)	Deformation	deformation	Deformation
110	1.95	-	-	1.64	-	-	4.98	-	-
120	2.13	-	-	1.79	-	-	5.45	-	-
160	2.83	-	-	2.38	-	-	7.25	-	-
170	3	-	-	2.54	-	-	7.7	-	-
180	3.18	-	-	2.68	-	-	8.16	-	-
190	3.36	-	-	2.83	-	-	8.61	-	-
240	-	4.25	-	-	3.58	-	-	9.46	-
250	-	4.43	-	-	3.73	-	-	9.85	-
320	-	5.67	-	-	4.77	-	-	12.05	-
340	-	6.02	-	-	5.07	-	-	13.34	-
350	-	6.2	-	-	5.22	-	-	13.79	-
430	-	7.61	-	-	6.41	-	-	16.94	-
620	-	-	10.91	-	-	9.25	-	-	21.56
630	-	-	11.15	-	-	9.4	-	-	22.61
700	-	-	12.39	-	-	10.44	-	-	24.4
720	-	-	12.73	-	-	10.74	-	-	25.1
780	-	-	13.8	-	-	11.63	-	-	27.19
800	-	-	14.15	-	-	11.94	-	-	27.88
970	-	-	17.17	-	-	14.47	-	-	33.81

Table 9 demonstrates significant variances between the three samples, particularly considering their brittleness, leading to shattering at the highest stress (DTS) levels. Remarkably, sample CGI exhibits higher bearing strength compared to RMGI and RC. Specifically, RC emerges as more than five times stronger than CGI, while RMGI is more than twice as strong as CGI based on numerical comparisons of the three samples. In materials science, 'deformation' refers to how applied forces can alter an object's size or shape. Interestingly, the deformation value of RC surpasses that of RMGI and CGI. This observation suggests that RC possesses greater resilience in deformation compared to the latter two, indicating its ability to tolerate higher stress levels.

## Discussion

Luting cements play a crucial role in fixed prosthodontics by securely bonding indirect restorations to prepared teeth. This attachment not only ensures the longevity of the restoration but also helps maintain the pulp vitality of natural abutments in fixed partial dentures, thereby restoring lost function [15]. To ensure satisfactory performance in luting applications, these cements must possess sufficient resistance to dissolution in the oral environment and establish a robust bond through a combination of mechanical interlocking and adhesion. High strength in compression and tension is essential for these cements to meet the necessary requirements [2].

In this study, three types of luting cement were utilized: CGI, RMGI, and RC. A notable advantage of glass ionomers lies in their ability to form bonds with teeth. This bonding mechanism involves a hydrogen bond between the carboxyl groups of polyacrylic acid and the calcium in the tooth. Furthermore, these materials exhibit a low coefficient of thermal expansion, similar to tooth structure, ensuring sustained bonding and facilitating the release of fluoride to the neighboring tooth [16].

Glass ionomer cements are hindered by their high initial solubility, limited strength, poor wear resistance, and susceptibility to water during setting. In response to these limitations, RMGI cements were developed. These cements address the issue of high solubility by bonding to the inorganic dentin through a linkage with the calcium ion present. Similar to glass ionomers, this bonding mechanism involves an acid-base reaction that occurs in an aqueous environment. RMGI cements offer a unique combination of the benefits of both glass ionomers and resins, including fluoride release, enhanced resistance to microleakage, strong adhesion to tooth structure, and reduced solubility compared to traditional glass ionomers. Their polymerization process begins before the acid-base reaction is complete, further reducing solubility and making them particularly effective in moist environments [17].

In this study, both glass ionomer luting cements were delivered in capsules, chosen to enhance standardization of the material powder/liquid proportion and facilitate a more accurate interpretation of the results. Previous studies have demonstrated that variations in the powder/liquid ratio may adversely affect the mechanical properties [18].

Compressive strength

Compressive strength is among the important factors for the success of fixation and is a critical indicator. A robust compressive strength is essential to withstand masticatory forces, defined as the stress at which a material fractures [3].

The findings of this recent study indicate significant variations in compressive strengths among the tested luting cements. The recorded values, ranked from highest to lowest, are: i) resin adhesive cement, ii) RMGI cements, and iii) CGI cement. Importantly, all luting materials surpassed the minimum required compressive strength level (50 MPa) established by ISO standard 9917.

The FEA results indicate that the compression stress of CGI is 101.08 MPa at a load of 810 N, RMGI is 161.73 MPa at a load of 1020 N, and RC is 348.29 MPa at a load of 2600 N. Importantly, the results obtained by FEA closely match the experimental results.

It is noteworthy that the results of this study are comparable to those of other published studies on the compressive strength properties of luting cements. For instance, the findings of Jefferies S et al. [18] demonstrated that RC exhibited higher compressive strength than RMGI cement, which aligns with the findings of this study.

The difference in compressive strength between resin luting cement and glass ionomer luting cement primarily stems from their distinct compositions and setting mechanisms [19]. Resin luting cement typically consists of resin monomers, fillers, and other additives. These cements polymerize through a chemical reaction, resulting in a strong and durable bond. The resin matrix provides high compressive strength, making it suitable for applications where load-bearing capacity is crucial, such as in the case of crowns and bridges [20]. In contrast, glass ionomer luting cement is a dental material containing glass particles and an acid-base reaction component. The setting reaction involves an acid-base reaction between an acidic component and a basic component. While glass ionomer cements exhibit good biocompatibility and bond well to tooth structure, they generally demonstrate lower compressive strength compared to resin luting cements [4].

Resin-based materials typically offer higher mechanical strength, whereas glass ionomer cements excel in ease of use, fluoride release, and biocompatibility. The decision between the two hinges on the specific clinical requirements and conditions for each dental application [19].

The mixing method plays a significant role in determining compressive strength. RC was packaged in dual-barrel syringes with single-use automix tips, ensuring thorough mixing of the two components. The components were completely mixed using a spiral mixer in the barrel syringes, minimizing the incorporation of additional air during the mixing process. Micro-pores, which are internal defects of the samples, directly impact the mechanical properties of the materials [20].

The CGI examined in this study displayed the lowest mean compressive strength value, consistent with findings from a study by Tavaner MS et al. [21]. It is hypothesized that this significant decrease in compressive strength may be attributed to its greater water sorption. The absorption of water can result in swelling, thereby weakening the material and reducing its mechanical properties, including compressive strength.

In this study, resin-modified glass ionomer cements (RMGICs) exhibited higher compressive strength compared to CGI cements (GICs), consistent with findings reported by Xie D et al. [22]. This improvement in strength can be attributed to several factors associated with the modification of the traditional glass ionomer formulation: RMGICs contain a resin component, which adds strength to the material. The resin provides a more rigid and durable matrix, contributing to enhanced mechanical properties. During the setting process, the resin component undergoes polymerization, creating a cross-linked network within the cement. This polymerization process results in a more stable and stronger structure compared to the chemical setting reaction observed in conventional GICs [23].

The liquid component of RMGICs introduces two significant concerns: Firstly, the presence of carboxylic acid groups (COOH), either directly or in close proximity to the acrylic backbone, can hinder the full conversion of carboxylic acid to carboxylate complexes. This interference may affect the formation of salt bridges and the migration of ions, potentially compromising the cement's fracture toughness and strength. Secondly, increasing the molecular weight and concentration of polyacids in the liquid component can offer advantages for enhancing the compressive strength of the cement [24].

RMGI cements (RMGICs) often exhibit lower water sensitivity compared to CGICs. This reduced water absorption helps maintain the material's integrity, preventing it from weakening over time [21]. Additionally, RMGICs typically demonstrate better adhesion to tooth structure and other dental materials. This improved bond strength enhances the overall performance of the restoration, contributing to its longevity and resistance to compressive forces [24].

Diametric tensile strength

DTS is an important mechanical property to assess, especially for cements prone to mechanical failure under tensile stress [15].

In the present study, Group RC exhibited the highest DTS, measuring 13.819. This was followed by Group RMGI, which had a DTS of 5.742, and Group CGI, which showed the lowest DTS at 2.652.

The FEA results indicated that sample RC exhibited higher stress strength (DTS) compared to RMGI and CGI samples, which was consistent with the experimental findings. Specifically, sample RC required a significantly higher stress force than RMGI and CGI samples to reach the breaking point.

The elevated DTS mean value of RC may stem from the composition of the resin-based restorative, where monomers, initiators, catalysts, and additives collectively form the reactive component. The robust mechanical characteristics and enduring stability can be ascribed to the blend of UDMA, aromatic aliphatic-UDMA, and PEG-400 DMA, which interconnect (cross-link) during polymerization. UDMA serves as the primary constituent of the monomer matrix, offering moderate viscosity and imparting robust mechanical attributes. The extensive cross-linked polymer structure contributes to the high DTS. Furthermore, the polymerization that precedes the completion of the acid-base reaction reduces the solubility of these products, making the material more advantageous in moist environments and more resistant to degradation compared to CGI [20].

Yoshida K et al. [25] demonstrated that resin luting cements, which assess the tensile strength of friable materials to avoid the difficulties inherent in flexural tensile strength tests, were markedly less soluble than conventional luting agents.

The limitations of the present study were: 1) The specimens were fabricated in a cylindrical shape according to ISO and ADA standards, meaning the effect of luting cement on anatomically shaped specimens and the actual thickness of the luting layer was not evaluated. 2) Only two mechanical properties, compressive strength and DTS, were assessed in this study.

## Conclusions

In conclusion, resin-based cements offer greater mechanical strength, whereas glass ionomer cements are advantageous in terms of ease of use, fluoride release, and biocompatibility. The choice between these cements should be guided by the specific clinical needs and conditions of each dental application.

## References

[1] Saran R, Upadhya NP, Ginjupalli K, Amalan A, Rao B, Kumar S (2020). Effect on physical and mechanical properties of conventional glass ionomer lutting cements by incorporation of all-ceramic additive: an in vitro study. Int J Dent.

[2] Patil SG, Sajjan MS, Patil R (2015). The effect of temperature on compressive and tensile strength of commonly used lutting cements: an in vitro study. J Int Oral Health.

[3] Al-Khadim AH, Abdullah H, Al-Ani ST (2018). Effect of thermocycling on the compressive strength of selected luting cements. IIUM Med J Malaysia.

[4] Sidhu SK, Nicholson JW (2016). A review of glass-ionomer cements for clinical dentistry. J Funct Biomater.

[5] Heboyan A, Vardanyan A, Karobari MI (2023). Dental luting cements: an updated comprehensive review. Molecules.

[6] Leung GK, Wong AW, Chu CH, Yu OY (2022). Update on dental luting materials. Dent J (Basel).

[7] Hutton DV (2004). Fundamentals of Finite Element Analysis. http://chrome-extension://efaidnbmnnnibpcajpcglclefindmkaj/https://wp.kntu.ac.ir/fz_kalantary/Source/Finite%20element%20method/Books-Numerical/Fundamentals%20of%20Finite%20Element%20Analysis,%20Hutton%20(2004).pdf.

[8] Dorado S, Arias A, Jimenez-Octavio JR (2022). Biomechanical modelling for tooth survival studies: mechanical properties, loads and boundary conditions-a narrative review. Materials (Basel).

[9] International Organization for Standardization (2007). Dentistry - Water-based cements: Part 1: Powder/liquid acid-base cements. https://www.iso.org/standard/45818.html.

[10] American Dental Association (1989). ANSI/ADA specification no. 66 for dental glass ionomer cements. Council on Dental Materials, Instruments, and Equipment. J Am Dent Assoc.

[11] Mazumdar P, Chowdhury D (2021). Manual of Laboratory Testing Methods for Dental Restorative Materials. https://www.wiley.com/en-us/Manual+of+Laboratory+Testing+Methods+for+Dental+Restorative+Materials-p-9781119687993.

[12] Cattani-Lorente MA, Dupuis V, Moya F, Payan J, Meyer JM (1999). Comparative study of the physical properties of a polyacid-modified composite resin and a resin-modified glass ionomer cement. Dent Mater.

[13] Cefaly DF, Franco EB, Mondelli RF, Francisconi PA, Navarro MF (2003). Diametral tensile strength and water sorption of glass-ionomer cements used in atraumatic restorative treatment. J Appl Oral Sci.

[14] Tarasachi V (2016). Patient specific 3D Finite element modelling analysis and verification of dental implant navigation and insertion system.

[15] Nischitha AB, Padmaja S (2023). Comparative evaluation of the diametral tensile strength of four commercially available luting cements: an in- vitro study. In J Inn Sci Res Technol.

[16] Anusavice KJ, Phillips RW (2003). Phillips' Science of Dental Materials. https://evolve.elsevier.com/cs/product/9781455757282?role=student.

[17] Rêgo HM, Butler S, Santos MJ (2022). Evaluation of the mechanical properties of three resin-modified glass-ionomer materials. Biomed Res Int.

[18] Jefferies S, Lööf J, Pameijer CH, Boston D, Galbraith C, Hermansson L (2013). Physical properties and comparative strength of a bioactive luting cement. Compend Contin Educ Dent.

[19] Maletin A, Knežević MJ, Koprivica DĐ, Veljović T, Puškar T, Milekić B, Ristić I (2023). Dental resin-based luting materials-review. Polymers (Basel).

[20] Li Y, Lin H, Zheng G, Zhang X, Xu Y (2015). A comparison study on the flexural strength and compressive strength of four resin-modified luting glass ionomer cements. Biomed Mater Eng.

[21] Tavaner MS, Jafarpur D, Bagheri R (2017). Evaluation of compressive strength and sorption/solubility of four luting cements. J Dent Biomater.

[22] Xie D, Brantley WA, Culbertson BM, Wang G (2000). Mechanical properties and microstructures of glass-ionomer cements. Dent Mater.

[23] Khalil R, Al-Shamma A (2023). Physicomechanical characterization of a novel resin-modified glass ionomer luting cement functionalized with a phosphate functional monomer. In J Dent.

[24] Dionysopoulos D, Gerasimidou O, Papadopoulos C (2022). Modifications of glass ionomer cements using nanotechnology: recent advances. Rec Prog Mater.

[25] Yoshida K, Tanagawa M, Atsuta M (1998). In-vitro solubility of three types of resin and conventional luting cements. J Oral Rehabil.

